# A Human Platelet Calcium Calculator Trained by Pairwise Agonist Scanning

**DOI:** 10.1371/journal.pcbi.1004118

**Published:** 2015-02-27

**Authors:** Mei Yan Lee, Scott L. Diamond

**Affiliations:** Institute for Medicine and Engineering, Department of Chemical and Biomolecular Engineering, University of Pennsylvania, Philadelphia, Pennsylvania, United States of America; University of Virginia, United States of America

## Abstract

Since platelet intracellular calcium mobilization [Ca(t)]_i_ controls granule release, cyclooxygenase-1 and integrin activation, and phosphatidylserine exposure, blood clotting simulations require prediction of platelet [Ca(t)]_i_ in response to combinatorial agonists. Pairwise Agonist Scanning (PAS) deployed all single and pairwise combinations of six agonists (ADP, convulxin, thrombin, U46619, iloprost and GSNO used at 0.1, 1, and 10xEC_50_; 154 conditions including a null condition) to stimulate platelet P_2_Y_1_/P2Y_12_ GPVI, PAR1/PAR4, TP, IP receptors, and guanylate cyclase, respectively, in Factor Xa-inhibited (250 nM apixaban), diluted platelet rich plasma that had been loaded with the calcium dye Fluo-4 NW. PAS of 10 healthy donors provided [Ca(t)]_i_ data for training 10 neural networks (NN, 2-layer/12-nodes) per donor. Trinary stimulations were then conducted at all 0.1x and 1xEC_50_ doses (160 conditions) as was a sampling of 45 higher ordered combinations (four to six agonists). The NN-ensemble average was a calcium calculator that accurately predicted [Ca (t)]_i_ beyond the single and binary training set for trinary stimulations (R = 0.924). The 160 trinary synergy scores, a normalized metric of signaling crosstalk, were also well predicted (R = 0.850) as were the calcium dynamics (R = 0.871) and high-dimensional synergy scores (R = 0.695) for the 45 higher ordered conditions. The calculator even predicted sequential addition experiments (n = 54 conditions, R = 0.921). NN-ensemble is a fast calcium calculator, ideal for multiscale clotting simulations that include spatiotemporal concentrations of ADP, collagen, thrombin, thromboxane, prostacyclin, and nitric oxide.

## Author Summary

Platelets regulate clotting during injury to prevent blood loss. Hyperactive platelets may increase risk of thrombosis, whereas hypoactive platelets may increase risk of bleeding. Platelets are activated during a clotting event by agonists, through different signaling pathways, all of which converge on intracellular calcium mobilization. Calcium mobilization is a global metric of platelet activation. Predicting platelet response to different combinations of agonists is essential to scoring bleeding or clotting risks or drug response. We collected pairwise agonist scanning data, in which platelets are activated by all single and pairwise combinations of six important agonists at low, medium and high doses, from 10 donors and subsequently trained artificial neural networks. The combined trained model was able to predict the dynamic calcium time traces of combinations of three, four, five and six agonists at various dose ranges, as well as conditions where agonists were added sequentially. The data-driven neural network model is computationally fast and is able to capture a significant level of signaling complexity within the human platelet.

## Introduction

Platelet activation during heart attack and stroke occurs through combined signaling pathways involving various receptors responding to collagen, thrombin, ADP, and thromboxane. Endothelial production of prostacyclin is highly protective against thrombotic platelet activation as revealed by the known cardiovascular risks of COX-2 inhibitors. Similarly, endothelial production of NO has many cardiovascular effects via vasodilation and platelet inhibition. The clinical importance of these pathways is seen in the number of drugs in clinical trials or approved that target GPVI signaling, thromboxane, ADP, or thrombin. More than 50 million U.S. adults take aspirin to inhibit platelet COX-1 production of thromboxane in order to reduce long-term risk of cardiovascular disease [1]. Clopidogrel antagonizes ADP activation of platelet P_2_Y_12_ receptors and is widely prescribed. Numerous anticoagulants are approved to target the generation or activity of thrombin.

**Fig 1:**
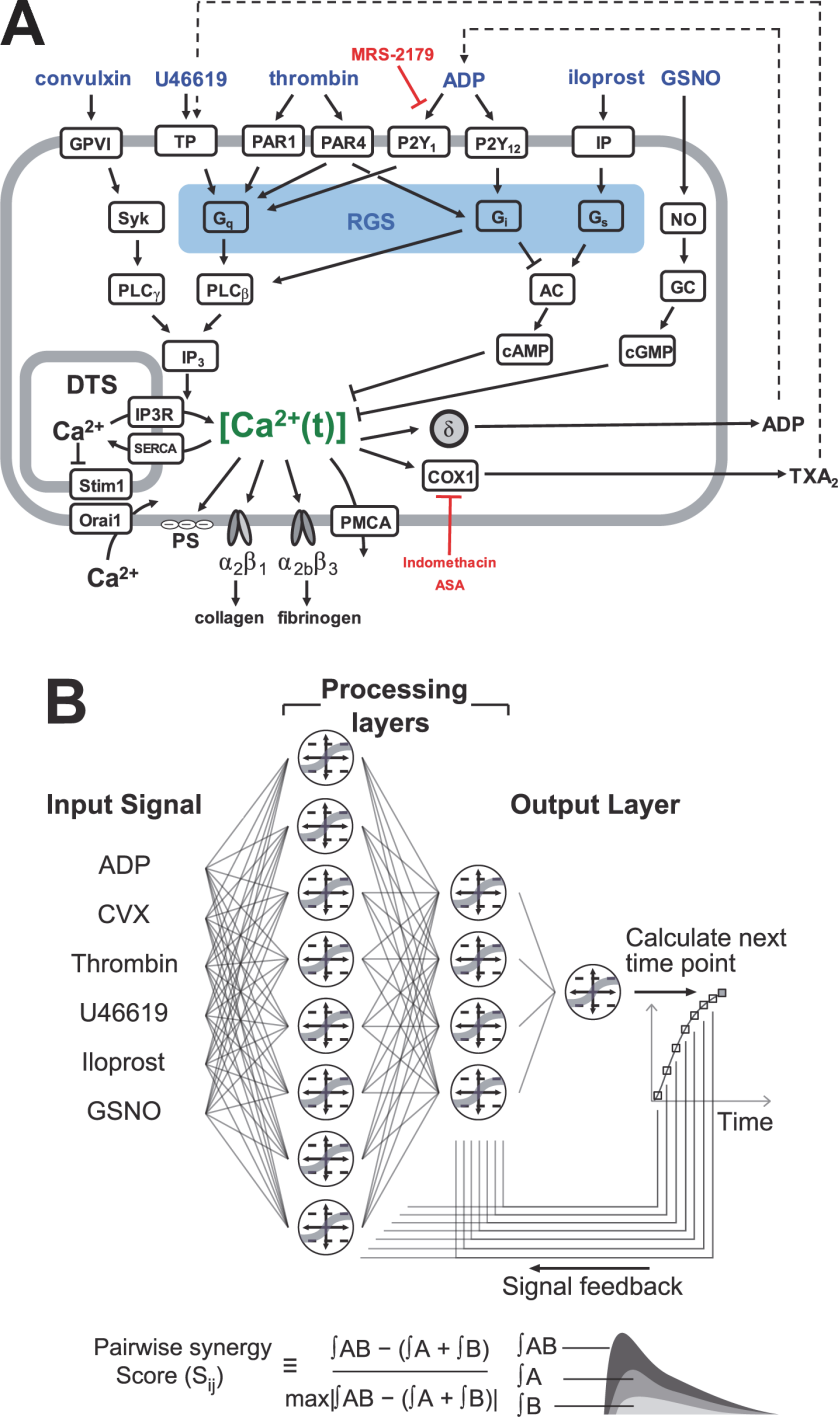
Platelet signaling pathway and neural network architecture. **(A)** The six agonists used in this study (ADP, convulxin, U46619, thrombin, iloprost, GSNO) and their respective platelet signaling pathways, all of which converge upon intracellular calcium mobilization. **(B)** A two-layer, 12-node neural network architecture was employed. Agonist concentrations at a given time point were fed into the processing layers; the layers then integrated the input signal with feedback at t = 1, 2, 4, 8, 16, 32, 64 and 128 seconds to calculate [Ca^2+^]_i_ at the next time point. The pairwise synergy score (Sij) was also defined as the difference in integrated calcium response to two agonists used together and integrated response to agonists used individually, normalized by the maximum synergy score in the experiment.

Platelet activation occurs through multiple signaling pathways in which agonists bind specific receptors on the platelet to trigger signaling in a dose-dependent manner. During a clotting episode, platelets respond to exposed surface collagen, released ADP, synthesized thromboxane, and the serine protease thrombin, all while being simultaneously modulated by endothelial derived nitric oxide and prostacyclin. These receptor-mediated signaling pathways are not independent and significant crosstalk can occur (**Fig. 1A**).

The Pairwise Agonist Scanning (PAS) method was first developed by Chatterjee et al. (2010) using EDTA-treated platelet rich plasma (PRP) to quantify and predict the interactions between multiple pathways (**S1 Fig.**) [2]. The PAS method measures platelet calcium responses to all individual and pairwise combinations of agonists at low, medium and high concentrations (154 conditions total for six agonists at 0.1, 1, and 10xEC_50_, including a null condition). Because EDTA chelates extracellular calcium and prevents store operated calcium entry (SOCE), the measured calcium data obtained using EDTA-treated PRP is enriched in the regulatory events surrounding IP_3_-mediated calcium release from the dense tubular system (DTS). With PAS data, Chatterjee et al. were able to train an artificial neural network (NN) to predict platelet calcium response to combinations of agonists beyond the training data, such as trinary combinations, sequential additions of agonists, and combinatorial responses of four to six agonists [2]. The NN model builds an estimate of higher order interactions (response to >2 agonists) by combination of the measured binary interactions. A metric called the pairwise synergy score was defined to quantify the extent of cross-talk between pairs of agonists (**Fig. 1B**) [2]. The pairwise synergy score (S_ij_) was defined to be the difference between the integrated area (area under the curve) for the combined response to agonists *i* and *j* relative to the integrated area for both the individual agonist responses added together, normalized to the maximum absolute S_ij_ [2]. In other words, the synergy score is a measure of deviation of the platelet response from the simple additive response of each agonist. A positive S_ij_ value indicates synergistic behavior between agonists *i* and *j* whereas a negative S_ij_ value indicates antagonistic or saturating behavior and S_ij_ = 0 represents a purely additive response.

Since many platelet pathways are triggered distally of IP_3_-released calcium and SOCE, the intracellular calcium concentrations can be used as a global metric of platelet activation. Calcium is the central “node” in platelet signaling, in that elevated calcium levels are central to downstream clotting events such as integrin activation, granule release, shape change, and phosphatidylserine exposure by platelets [3–5]. The ability to predict dynamic calcium traces for combinations of agonists enables the targeting of specific platelet pathways to increase or decrease platelet activity so as to achieve desired clinical outcome. NN trained on PAS data provides accurate calcium responses to different dose combinations of important agonists and is essential for simulating platelet function under flow. As an in silico predictor of calcium regulation, the NNs trained by PAS can be embedded in multi-scale simulations of platelet deposition under flow conditions. In the work of Flamm et al. (2011) [6], NN were trained via PAS using calcium-containing PRP and then used to predict platelet deposition rates on collagen in the absence of thrombin by accounting for platelet signaling in response to laboratory analogs of collagen, ADP, thromboxane, and prostacyclin.

A universal platelet calcium calculator provides a reference for platelet function of a healthy human. Platelet gain of function or loss of function in patients can therefore be measured in a high dimensional approach using the PAS method. Furthermore, since the specific pathways in the platelet that contribute to platelet gain or loss of function can be identified by PAS as well, PAS can be used to predict the sensitivity and resistance of drugs that target those specific agonist pathways, even loss of function mutations have been discovered with PAS [6]. Additionally, the calcium calculator can be embedded into a multiscale simulation of clotting under defined hemodynamic conditions.

In the current study, the PAS method was expanded for the use of exogenously added thrombin in the presence of normal calcium and included the potent platelet inhibitors iloprost and nitric oxide (NO). Thrombin is an extremely potent platelet activator via cleavage of platelet PAR1 and PAR4 receptors. Additionally, to estimate average healthy human platelet responses, calcium data was obtained from 10 healthy donors as an ensemble-averaged predictor of platelet calcium. Distinct from the prior PAS in Chatterjee et. al (2010) where PAR1 and PAR4 agonist peptides were used with platelet rich plasma (PRP) treated with EDTA, the current study required a means to study exogeneously added thrombin without endogenous production of thrombin in PRP with normal calcium. In the current experimental design, blood was drawn into 250 nM apixaban (K_i_ = 0.08 nM) [7], a direct Factor Xa inhibitor, which does not alter extracellular calcium levels but prevents endogenous thrombin generation. This assay therefore allows the contribution of SOCE and includes the signaling distal of thrombin proteolytic activity on PAR1 and PAR4. Furthermore, the NN-ensemble method was employed to increase accuracy and robustness in NN predictions.

## Materials and Methods

### Ethics Statement

Whole blood was drawn by venipuncture from healthy donors according to the University of Pennsylvania Institutional Review Board guidelines (protocol number: 805305), into a syringe containing apixaban (SelleckChem) with a final concentration of 250 nM. Donors self-reported to be free of any medications or alcohol use for three days prior to the blood draw. Female donors self-reported to not using oral contraceptives.

### Pairwise Agonist Scanning (PAS)

Platelet rich plasma (PRP) was then obtained by subjecting the whole blood sample to centrifugation at 120g for 12 minutes. Then, 2 ml of PRP was incubated with a vial (single microplate size) of Fluo-4 NW dye mixture (Invitrogen) reconstituted with 7.8 ml of HEPES buffer and 200 μL of 77 mg/ml reconstituted probenecid (Invitrogen) for 30 minutes [2]. All single and pairwise combinations of six agonists (ADP, convulxin, thrombin, U46619, iloprost and GSNO) at low, medium and high concentrations (0.1, 1, and 10X EC_50_), as well as a buffer condition (154 conditions total x 2 replicates) were dispensed into a 384-well plate (called the ‘agonist plate’) using a high throughput liquid handler (PerkinElmer Janus). The PAS agonists were: ADP (P_2_Y_1_/P_2_Y_12_ activator, EC_50_ = 1 μM), convulxin (GPVI activator, EC_50_ = 2 nM), thrombin (PAR1/PAR4 activator, EC_50_ = 20 nM), U46619 (TP activator, EC_50_ = 1 μM), iloprost (IP activator, EC_50_ = 0.5 μM) and GSNO (NO donor, EC_50_ = 7 μM) (**S2 Fig.**). ADP and GSNO were obtained from Sigma-Aldrich, convulxin from Pentapharm, thrombin from Haematologic Technologies Inc., U46619 and iloprost from Tocris Bioscience. After incubation with dye, the PRP was dispensed into a 384-well plate (called the ‘read plate’). Both the agonist and read plate were loaded into a Molecular Devices FlexStation 3, a fluorescence reader with auto-pipetting capabilities. Agonists were dispensed to a column of wells containing the PRP, where well fluorescence F(t) was read and normalized by the pre-dispense baseline. Specifically, 20 μL of agonist was added to 30 μL of PRP in each well, giving a final volume of 50 μL. In each well, the final concentration of PRP after agonist addition was 12% PRP by vol., and the volume of calcium dye was 15 μL (30% dye by vol.). Readings were taken in intervals of 2.5 seconds. The fluorescence was read for 20 seconds before dispense, and readings were taken for 210 seconds after each dispense (EX/EM, 485 nm/525 nm). The entire plate was read, column-wise, in under 90 minutes. PAS was conducted on PRP from ten donors (50% male), each in replicate on two different days (20 PAS experiments total). In separate tests using indomethacin (Sigma-Aldrich) to block COX1 and apyrase (Sigma-Aldrich) to degrade released ADP, there was no evidence for autocrine signaling in the dilute PRP conditions of the experiment (**S3 Fig.**), as previously found with EDTA-treated PRP [2].

### Trinary, Higher Order Combinations and Sequential Addition Experiments

In experiments with trinary mixtures of agonists, all single and trinary combinations of the same six agonists at two different concentrations (0.1x and 1x EC_50_), as well as a null buffer condition (173 conditions total x 2 replicates) were prepared in the agonist plate. This experiment was done once for each of the 10 PAS donors. There are 3,402 possible conditions involving four, five, or six agonists (higher order combinations of agonists) at low, medium and high concentrations. The higher order combination space was sampled in equal proportions (approximately 1.3% each of 4 to 6 agonist conditions). Thus, a total of n = 45 higher order combinations were sampled (16 four-agonist, 19 five-agonist, and 10 six-agonist conditions). These experiments were done seven times spanning five donors (two repeat experiments for two of the donors), neither of which were present in the PAS training set. In the sequential addition experiments, all conditions involving sequential addition of three agonists (ADP, convulxin and U46619) at three different concentrations and a null buffer condition (55 conditions total x 2 replicates) were prepared in the agonist plate. The sequential addition experiment was done once on a single donor.

### Calculation of Synergy Scores

The pairwise synergy score (S_ij_) was defined to be the difference between the integrated calcium for the combined response to *ij*-agonists and the sum of the integrated calcium for both the individual agonist responses used independently, normalized by scaling to the maximum absolute synergy score observed in the experiment (**Fig. 1B**) [2]. Synergy scores range from -1 to 1 (positive, synergistic; 0, additive; negative, antagonistic). Trinary synergy scores (S_ijk_) were also similarly calculated as the difference of the combined response to *ijk*-agonists from the response for all three individual agonist responses. In general, synergy scores (S_n_) are defined by Eq. 1, where the variable A_i_ represents the integrated calcium for the response to agonist i used independently, and A_1…n_ is the area under the curve for the response to agonists 1 through n used simultaneously (n = 6 maximum for the six agonists deployed).

$$S_{n}=\frac{A_{1\ldots n}-\sum_{i=1}^{n}A_{i}}{\max|A_{1\ldots n}-\sum_{i=1}^{n}A_{i}|}$$ 1

### Neural Network Training and Averaging

The replicate wells in each PAS experiment duplicate were averaged before training. A 2-layer, 12-node dynamic neural network (NN), as employed in Chatterjee et al. (2010), was trained on each averaged PAS experiment 10 times (n = 100), each time with a different set of initial weights and randomized division of 154 single and pairwise time course data into training and validation sets (90%/10%) (**Fig. 1B**). All neural network model construction and training were done using the MATLAB Neural Network Toolbox (MathWorks).

Training on a NN was done for a maximum of 1000 epochs, where each epoch was one pass through the training set followed by testing of the validation set. The training set vectors were used to optimize the NN weights and the validation set was used to test the weights during training so as to ensure the NN did not over fit to the training set. Early stopping was also employed, in which training would stop if the validation set error did not improve after five epochs. At the end of the training of each NN, the optimized NN weights would typically match the predicted time series to the experimental time series with a mean squared error anywhere on the order of 10^–4^ to 10^–2^.

**Fig 2:**
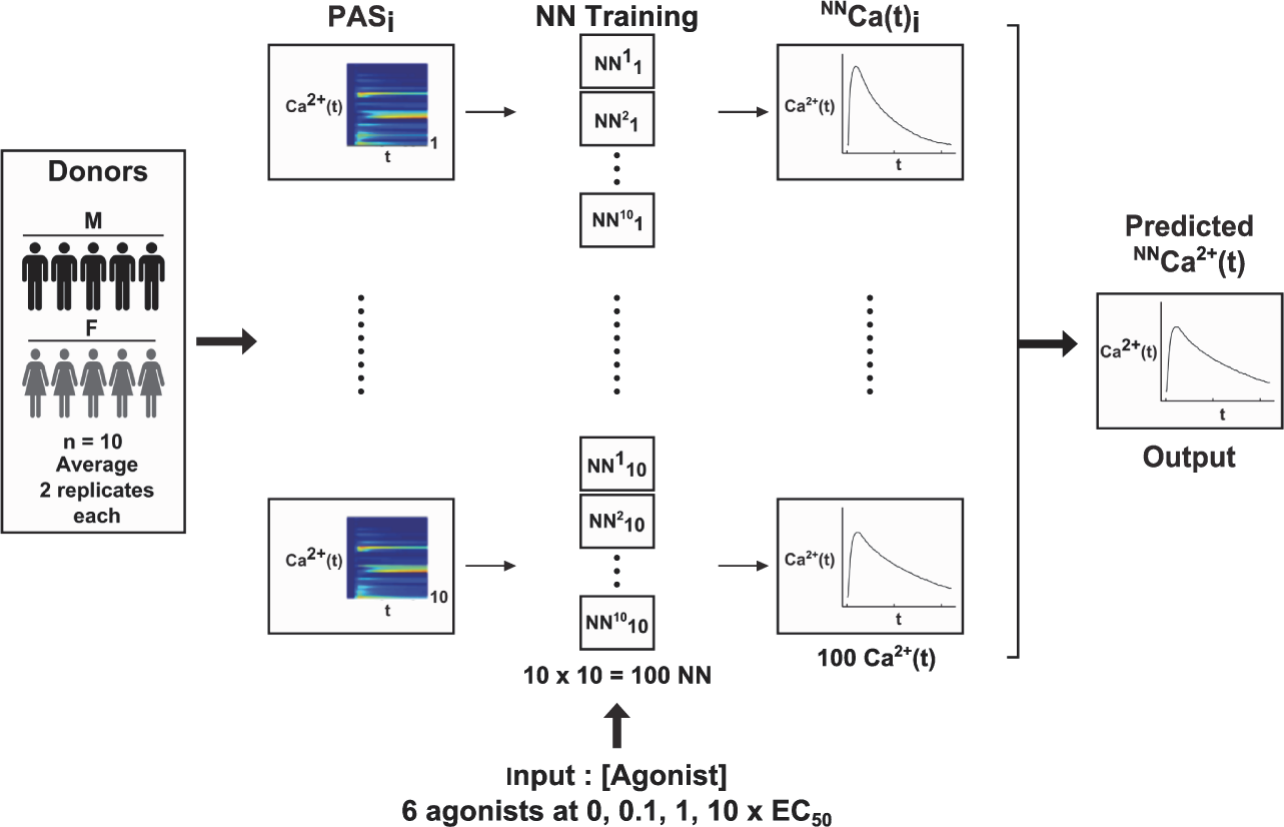
Experimental and computational workflow. The dataset consists of PAS experiments done in duplicate on PRP donated by ten donors (five male, five female). The duplicate experiments were averaged before training, i.e. n = 10. Each PAS experiment comprised a set of 154 calcium time traces, responses to all combinations of six agonists at three different concentrations and a buffer condition. A neural network was trained on each of the average PAS experiments giving a total of n = 100 trained neural networks. A different set of assay conditions (in this case, trinary combinations of the same six agonists at low and medium concentrations) are then input to each of the 100 trained neural networks (collectively called the ensemble NN) to generate predictions, which are then averaged and compared to the experimental average calcium time traces. These trinary combination experiments were done once each on the same ten donors, i.e. n = 10.

Each of the 100 trained NNs was then given the trinary experiment agonist concentration inputs and the resulting calcium time trace predictions were averaged to give an overall prediction for the average trinary experiment platelet response. The resulting synergy scores were also calculated and compared to the actual synergy scores for the trinary experiments. Similarly, each of the 100 trained NNs were given the higher order combination and sequential addition experimental concentration inputs, and the resulting calcium time trace predictions were compared to the measured values. A summary of the experimental and computational workflow is shown in **Fig. 2**.

In the testing of NN predictive abilities, output from all 100 NNs were averaged to give a final prediction. This approach is an ensemble method [8]. Ensemble methods are commonly used to overcome the inherent instability problem with NNs [8]. NNs (along with decision trees, multivariate adaptive regression splines etc.) are inherently unstable in that small differences in training data or conditions (e.g. initial weights) may cause variations in final predictions. Generating an ensemble of NNs and combining their outputs to produce a single prediction has been proven by Cunningham et al (2000) to be a robust solution to this problem [8].

In the testing of the NN on higher order combination experiments, two additional donors (one male, one female) were used in the training set (12 donors for a total of 120 NNs). None of the 12 donors used in the training set were present in the testing donor set (five donors for a total of seven experiments). The success of the higher order combination predictions suggests that the ensemble NN was sufficiently robust to predict outcomes of donor aggregates without their donor-specific PAS data during training, and that the NN ensemble has learned calcium mobilization patterns of an average healthy human. Furthermore, the sequential addition experiment predictions points to the ability of the ensemble NN to predict the outcome of an individual donor, not just aggregate outcomes of donors, and without having PAS data of that specific testing donor during training.

## Results

### Neural network prediction of platelet responses to binary agonist stimulation

**Fig 3:**
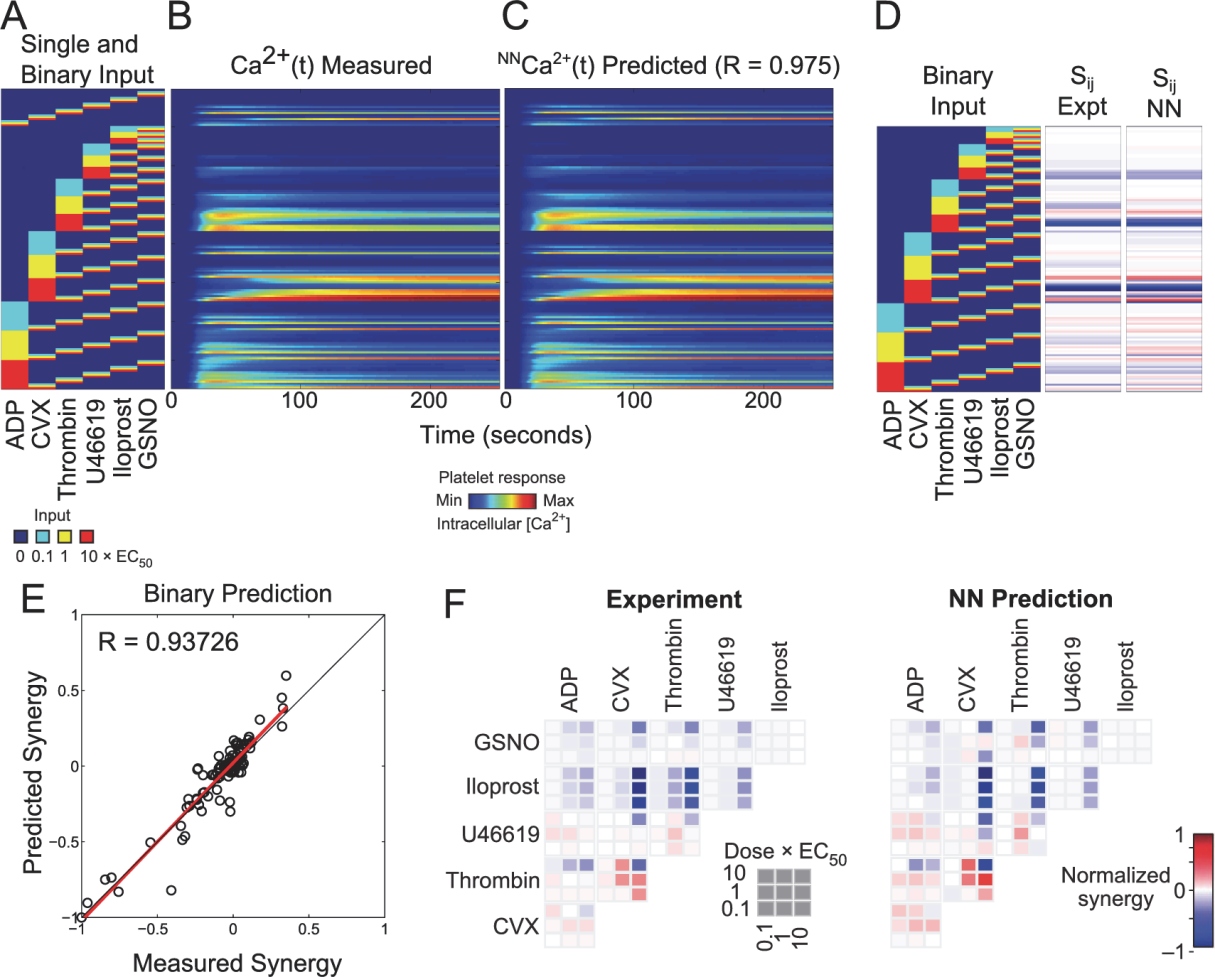
Neural network fit of PAS experiments. **(A)** PAS experiment input conditions: all single and pairwise combinations of six agonists at low, medium and high concentrations. **(B)** Measured average calcium time courses of ten donors in the PAS experiments. **(C)** The neural network trained on the PAS experiments of ten donors was able to fit the measured average pairwise calcium traces of those ten donors with a correlation coefficient of R = 0.975. **(D)** The experimental and NN-predicted 135 pairwise synergy scores for the PAS experiment. **(E)** The neural networks were able to fit the measured average pairwise synergy scores of those ten donors with a correlation coefficient of R = 0.937. **(F)** The experimental and NN-predicted synergy scores arranged by dose and agonist pairs.

The 10x10 NN-ensemble was trained on the pairwise agonist scanning (PAS) experiments of 10 donors and predicted the measured average pairwise calcium traces of those donors (**Fig. 3A-C**) with a correlation coefficient of R = 0.975 (**Fig. 3C**). **Fig. 3A** indicates all 154 single and pairwise conditions used in the PAS with the corresponding calcium time traces (**Fig. 3B**) and NN-predicted calcium time traces (**Fig. 3C**). From **Fig. 3A-C**, the calcium response to convulxin rose slowly, but became highly elevated and was sustained. In contrast, calcium responses to ADP or U46619 displayed rapid onset but were weaker and more transient than calcium responses to convulxin and thrombin. Interestingly, the thrombin response displayed rapid calcium mobilization, prominent elevation, and was more sustained than calcium responses observed in earlier studies with PAR1 and PAR4 agonist peptides [2]. A total of 135 binary synergy scores (all pairs of six agonists at three concentrations) for the PAS experiment and the NN-prediction are shown in vector form (**Fig. 3D**), representing the average human platelet phenotype. Both experimental and NN-predicted synergy values were plotted in heat map form in **Fig. 3D**. Similar to the time series prediction, the NN ensemble was able to predict the measured average pairwise synergy scores of those ten donors (R = 0.937) (**Fig. 3E**). Many synergy values clustered around the center of the range (S_ij_ ∼ 0, additive) with slightly more negative values extending to full antagonism (S_ij_ ∼ -1). The maximum synergy score did not exceed 0.5. The most negative synergies were found with pairwise mixtures that included iloprost. The experimental and NN-predicted synergy scores were also arranged by dose and agonist pairs (**Fig. 3F**). From **Fig. 3F**, iloprost was inhibiting for all agonists used in combination with it. GSNO was also inhibiting for most conditions, however, low dose GSNO slightly potentiated thrombin-induced calcium response. The combination of medium or high dose thrombin with medium dose convulxin was particularly synergistic, consistent with several findings [9,10]. Also, thrombin signaling was synergistic with thromboxane signaling which is a novel observation since both agonists signal through G_q_.

### Neural network prediction of platelet responses to trinary agonist stimulation

**Fig 4:**
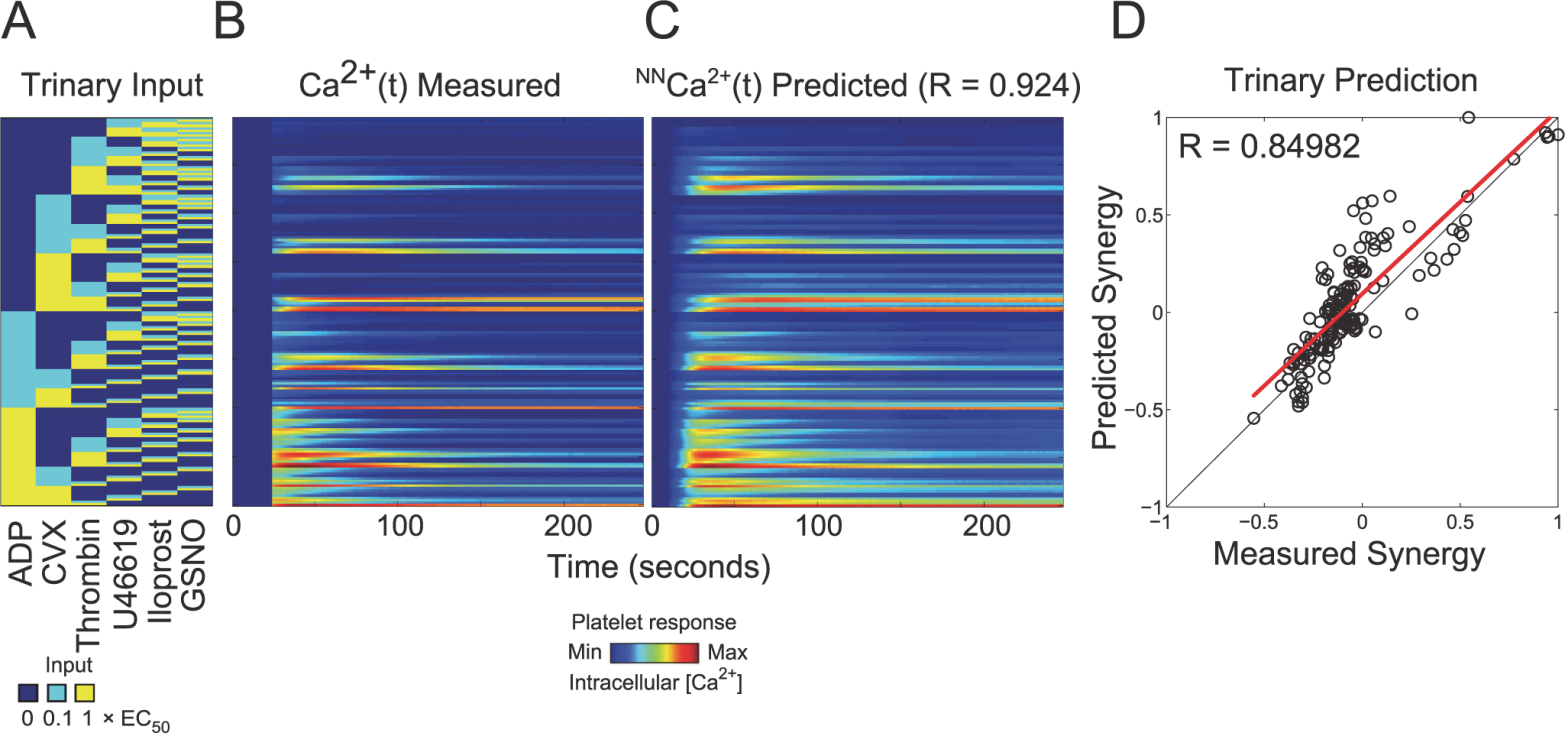
Neural network prediction of trinary experiments. **(A)** Trinary experiment input conditions: all trinary combinations of six agonists at low and medium concentrations. **(B)** Measured average calcium time courses of ten donors in the trinary experiments. **(C)** The neural network trained on the PAS experiments of ten donors was able to fit the measured average trinary experiment calcium traces of those ten donors with a correlation coefficient of R = 0.924. **(D)** There are 160 trinary synergy scores for the trinary combination experiment. The neural networks were able to fit the measured average trinary synergy scores of those ten donors with a correlation coefficient of R = 0.84982.

**Fig 5:**
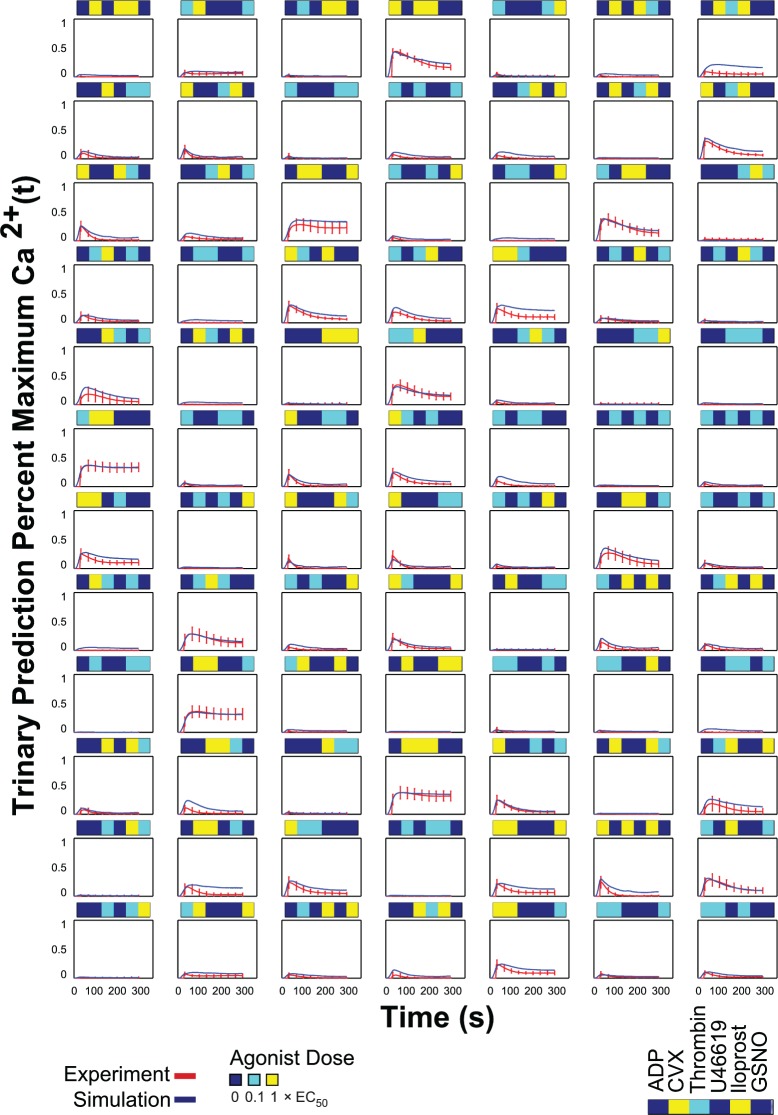
Examples of NN-predicted and measured calcium time traces for the trinary combination experiment. Plots of 84 of the 160 conditions in the trinary combination experiment (all trinary combinations of agonists at two concentrations: 0.1x EC_50_ and 1x EC_50_).

To test the predictive capability of the NN-ensemble beyond the training set, the NN-ensemble was used to predict the calcium output of trinary agonist stimulations. To avoid saturation effects of agonist induced signaling, the trinary combination experiments comprised all trinary combinations of six agonists at only the low and medium doses (**Fig. 4A**). The NN ensemble trained only on the PAS experiments of ten donors in duplicate was able to predict the measured average trinary calcium traces of those donors with high accuracy (R = 0.924) (**Fig. 4B-C**). This demonstrated the *de novo* predictive capability of the neural network model. There were 160 trinary synergy scores for the trinary experiment consisting of all trinary combinations of six agonists at two different concentrations (0.1 and 1x EC_50_). The NN trained only on the PAS experiments of ten donors in duplicate was able to predict the measured average trinary synergy scores of those ten donors with a correlation coefficient of R = 0.850. (**Fig. 4D**). The synergy scores plotted for the trinary experiments in **Fig. 4D**, though also clustered around zero, extended more toward 1 (synergistic) compared to the binary synergy scores (**Fig. 3E**), which extended toward -1 (antagonistic). This is expected, in part, because the trinary experiments were sampled across only the low and medium dose ranges, thus, there were fewer instances of saturation due to platelet activation by high doses of multiple agonists. Furthermore, only low and medium doses of the inhibitors iloprost and GSNO were used, so their strongest inhibitory/antagonistic effects at 10x EC_50_ were not present in the trinary data (but were present in the binary experiments). To illustrate the predictive power of the NN-ensemble, the full time series plots of a random sampling of the 160 trinary conditions are shown in **Fig. 5**. Full time series plots of all 160 trinary conditions rescaled to 0.5 are also shown in **S6 Fig.**. For the trinary stimulations, the predicted calcium time traces fit the experimental data over the full time domain with remarkable accuracy.

The trinary agonist experiments embed information about platelet signaling during in vivo hemostasis, thrombosis, or bleeding. For example, during the early stages of vessel wall injury, platelets are activated by collagen of the damaged vessel wall [11] which can also generate thrombin via the extrinsic pathway (distal of tissue factor). Concomitantly, endothelium-derived nitric oxide and prostacyclin modulate platelet functions. Therefore at this early stage the platelet is mainly exposed to these three agonists: exposed collagen, prevailing nitric oxide and prostacyclin, while thrombin is dynamically generated. Soluble agonists such as ADP and thromboxane become critically important during platelet mass build-up (sometimes called secondary aggregation) when activated platelets release ADP from dense granules and generate thromboxane via COX-1. Recent in vivo and in vitro studies reveal that the platelets in the “core” are exposed to high levels of thrombin, while the outer shell of platelets see little thrombin but are especially sensitive to the presence of thromboxane [12–16].

### Neural network prediction of higher order combination experiment

**Fig 6:**
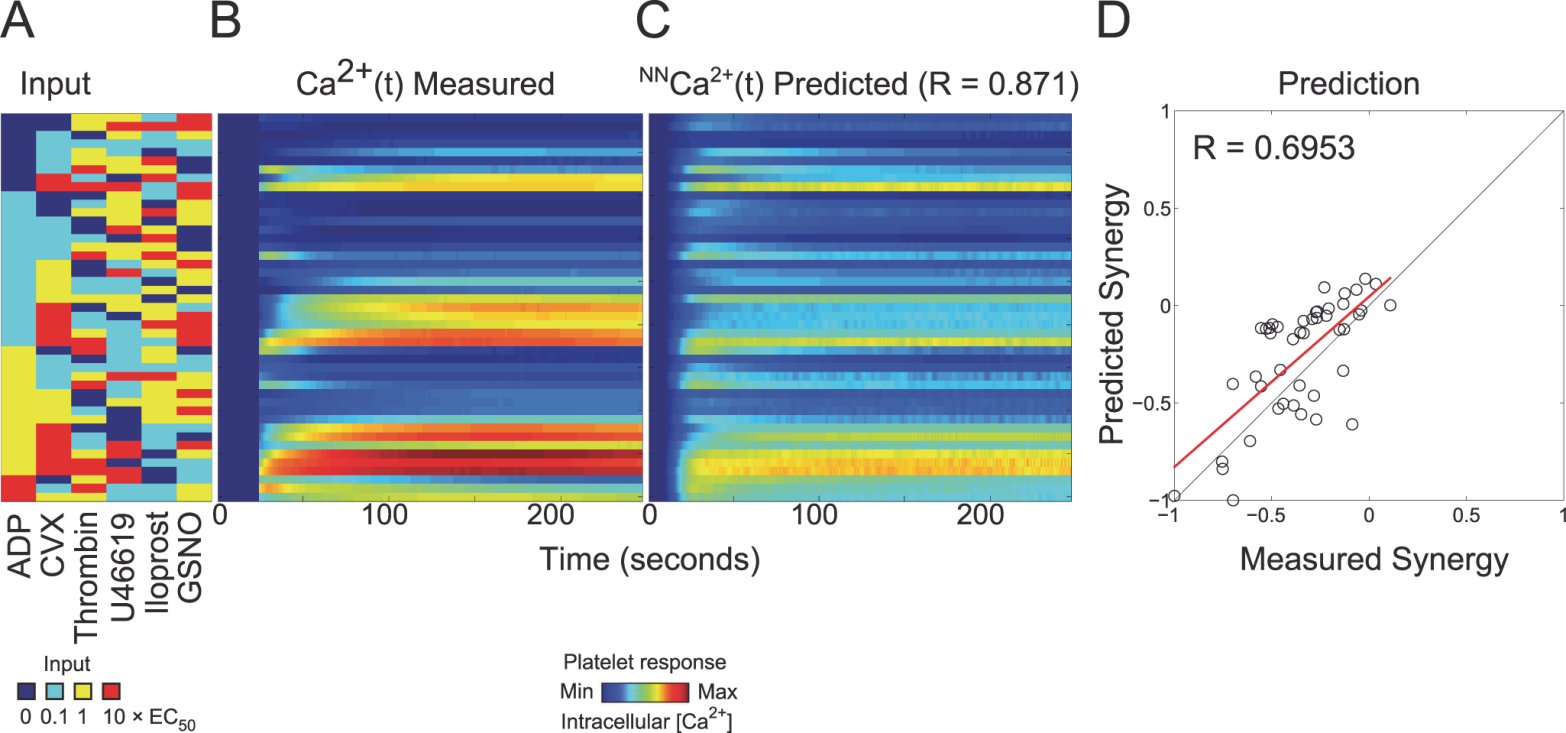
Neural network prediction of higher order combination experiments. **(A)** Experimental input conditions that are a random sample (n = 45) of the complete experimental space involving four, five, and six agonists at low, medium and high concentrations (n = 3,402). **(B)** Average measured calcium time courses of seven experiments spanning five donors in the higher order combination experiment. **(C)** The neural network trained on the PAS experiments of 12 donors was able to fit the measured experimental calcium traces with a correlation coefficient of R = 0.871. **(D)** The neural networks were able to fit the measured average synergy scores of those ten donors with a correlation coefficient of R = 0.6953.

**Fig 7:**
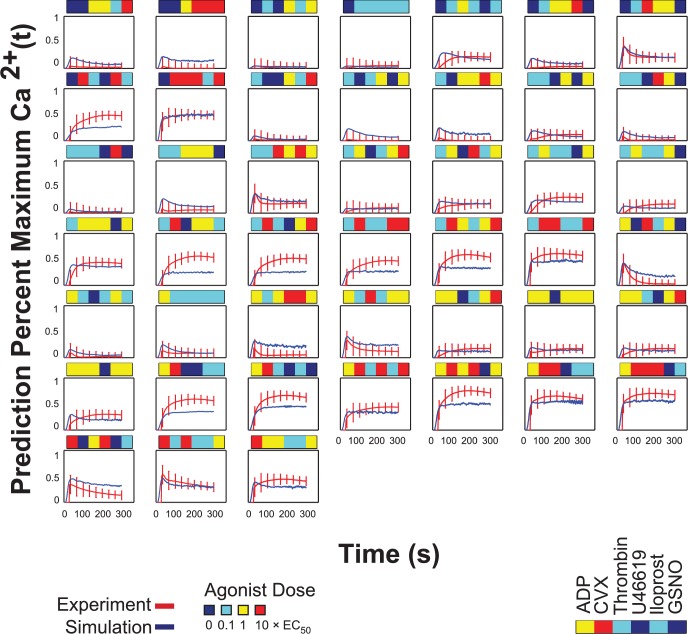
Calcium time traces for the higher order combination experiment. Plots of the 45 conditions in the higher order combination experiments.

The higher order test of the NN-ensemble comprised a 45-condition sampling of the full experimental space in equal proportions (n = 45 total combinations: 16 four-agonist, 19 five-agonist, and 10 six-agonist conditions) (**Fig. 6A**). In this subsequent higher order experiment, the donor pool for the generation of the PAS training dataset was expanded to 12 individuals, 10 of which were also previously used in the prediction of trinary combination outcomes. The higher order experiments were an aggregate of seven experiments spanning five donors, none of whom were utilized in the PAS training dataset. The NN-ensemble trained only on the PAS experiments of 12 donors was able to predict the calcium traces of the higher order combination experiments with sufficiently high accuracy (R = 0.871) (**Fig. 6A-C**). This higher order experiment represented a most challenging test of the *de novo* predictive capability of the neural network model, more so than the trinary combination experiments of **Fig. 4**. With up to six stimuli present, this experiment triggers an extraordinary range of signaling complexity in the platelet. The NN-ensemble trained only on the PAS experiments was able to predict the measured synergy scores with a correlation coefficient of R = 0.6953 (**Fig. 6D**). Compared to the synergy scores of the binary (**Fig. 3E**) and trinary experiments (**Fig. 4D**), the synergy scores of the higher order combination experiments tended to be more antagonistic due to saturation effects. Many of the measured and predicted higher ordered synergy scores were additive (S∼0) with none being highly synergistic (all S < 0.25) which occurs for saturated signaling by only a few of the agonists in the mixture. During saturation, a maximal amount of calcium is released by IP_3_ or conveyed by SOCE. Therefore, the actual calcium mobilization caused by high doses of ≥4 activating agonists was not expected to exceed the sum of calcium release due the individual agonists. The time series plots of all 45 conditions in the higher order combination experiment (**Fig. 6C and Fig. 7**) indicated that the NN-predicted time calcium time traces tended to consistently under predict calcium traces involving high dose convulxin. This may be because the NN had not been trained on any data that involves calcium levels as high as that triggered by combinations of 4–6 agonists including a high dose of the potent activator convulxin. Another theory is that the NN may have over predicted the saturation effects in calcium responses that may result from combinations of multiple agonists in addition to high dose convulxin. Furthermore, the NN predictions underestimated the effect of iloprost and GSNO, in that combinations that involved those agonists tended to have calcium level predictions that were higher than the experimental values. However, the overall shape of almost all the predictions fits the experimental time traces rather well, indicating that the NN ensemble was able to capture the kinetics of these higher order combination experiments. There was no apparent trend in calcium trace prediction accuracy with increasing number of agonist (four-agonist conditions had R = 0.82332, five-agonist conditions had R = 0.90699, six-agonist conditions had R = 0.79398). However, this sampling of 45 conditions was only 1.3% of the complete experimental space of 3,402 possible conditions.

### Neural network prediction of sequential addition combination experiment

**Fig 8:**
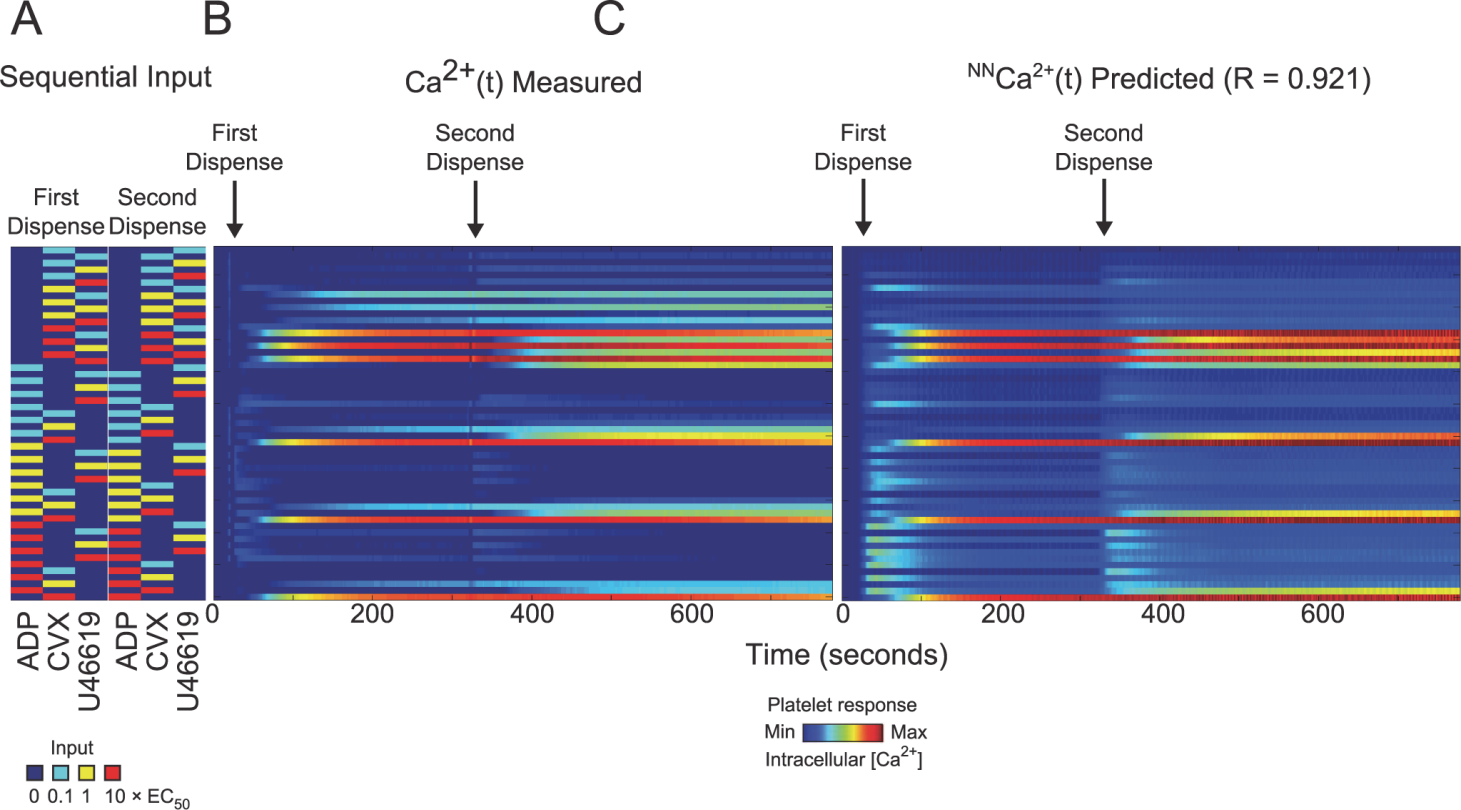
Neural network prediction of sequential addition experiments. **(A)** Experimental sequential input conditions (n = 54) for three agonists (ADP, convulxin and U46619). **(B)** Measured calcium time courses of Donor Y in the sequential addition experiment. **(C)** The neural network trained on the PAS experiments of ten donors was able to predict the measured sequential addition calcium traces of Donor Y with high accuracy (correlation coefficient, R = 0.921).

**Fig 9:**
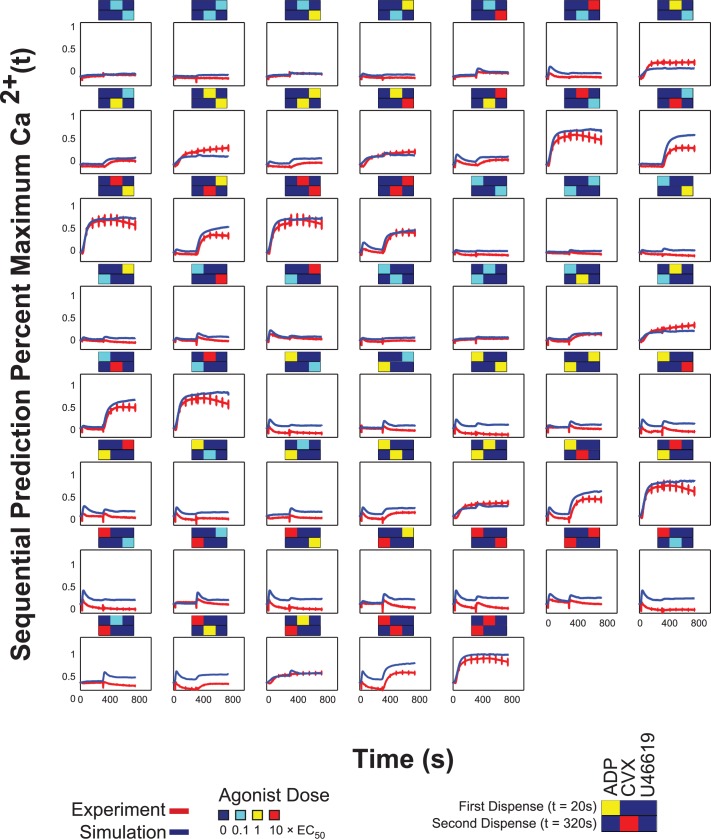
Calcium time traces for the sequential addition experiments. Plots of the 54 conditions in the sequential addition experiments.

A sequential addition experiment was done with all permutations for a two-dispense experiment of three agonists (ADP, convulxin and U46619) in the full dose range, i.e. 54 conditions total (**Fig. 8A**). Calcium time traces were plotted in heat map form in **Fig. 8B**; the arrows indicate the time at which the corresponding agonists in **Fig. 8A** were dispensed. The NN trained on the PAS experiments, in which agonist pairs were added simultaneously, was also able to predict the calcium output of the sequential addition experiment with a correlation coefficient of R = 0.921 (**Fig. 8C**). The plots of both the experimental and predicted time series are shown in **Fig. 9**.

Due to limitations of working with isolated platelets in vitro (requiring use within 3 hr from venipuncture), the PAS calcium readings for sequential addition tests required a 260 sec window per column for the 24 columns of the 384-well plate. The shorter 260 sec training interval still provided accurate predictions for the whole 780 sec window because many of the PAS calcium traces following agonist-mediated increases had decayed to resting levels within the 260 sec training interval. This was true of all agonist combinations except for those involving convulxin, which generated sustained calcium levels in the 260 sec window. Within this training interval, the PAS had captured the full kinetic effects of all the agonists except for convulxin. Extending the training window to 300 sec, where the second dispense in the sequential addition experiment had happened, would not confer additional information to the NN because convulxin responses would not have started decaying at the end of 300 seconds. Therefore, the NN trained on the PAS equipped with this kinetic information, in addition to crosstalk information between these six agonists from the pairwise conditions, was able to predict calcium responses to two agonists added sequentially with sufficiently high accuracy (R = 0.921), with a tendency for mild over prediction of calcium compared to the experimental time series. Nonetheless, the shape of the NN and measured calcium traces were quite similar (**Fig. 9**). As expected, in conditions where convulxin was added first in the sequential addition experiments, the NN predicted a sustained calcium level instead of a slight decay in calcium response toward the end of the 780 sec window, since this decay had not been captured in the 260 sec window of the PAS experiment used in NN training.

### Analysis of synergy scores

The synergy scores (S_ij_) reduced more than 25,000 calcium time points from a PAS experiment to a 135-parameter vector. Each synergy score is the most succinct first order measure of crosstalk between specific pairs of agonists at specific doses. The discovery of new synergistic and antagonistic effects between specific combinations of agonists via synergy scores can motivate efforts to study the underlying biochemical mechanisms (e.g. the thrombin-thromboxane positive synergy is unexpected since both signal through G_q_). Furthermore, the synergy score may underlie a drug risk (as seen with COX-2 inhibition therapy [2]), a patient-specific drug sensitivity or resistance. Future bottom-up models that predict the 135-parameter synergy vector may require interactions and pathways not explicitly represented in the currently prevailing platelet signaling model of **Fig. 1A**.

Synergy scores of the 10-donor, 20-experiment averaged PAS experiments were further analyzed to gain insight into platelet signaling. The same was done for the averaged trinary combination experiments and the higher order combination experiments. The mean of the standard deviation between donors for a given synergy score in the experiment is 0.0932 for binary dataset, 0.1722 for the trinary dataset, and 0.2029 for the higher order dataset. This reflects the variation in a given synergy score between donors. As the number of agonists involved increased, signaling complexity increased, resulting in larger donor variations in a given synergy score.

The mean of the pairwise S_ij_ was very close to zero (S_ij_ = - 0.0626), and the maximum synergy score was 0.3461. The mean of the trinary synergy scores was slightly less negative (S_ijk_ = -0.0401), and the maximum synergy score was 1, meaning that the maximum absolute synergy score is a synergistic one. Because trinary conditions were only sampled at the low and medium dose, calcium saturation from multiple high doses of agonists was avoided, therefore the synergy scores tended to be more synergistic. The mean of the higher order combination synergy scores was quite antagonistic (S = -0.3484), and the maximum synergy score was also very small (S = 0.1085). As more agonists are involved, platelet signaling tends to reach saturation, which shifts the mean synergy score towards antagonism, and lowers the maximum synergy score.

For the PAS and higher order combination experiments, the synergy score with the maximum magnitude was antagonistic (i.e. minimum synergy score is -1 for binary and higher order experiment averages) because iloprost and GSNO are both strong inhibitors in this assay. For the average PAS experiment, the most antagonistic synergy score involved a high dose of iloprost; for the higher order experiment, it involved low dose iloprost and high dose GSNO. The most antagonistic synergy score for the trinary experiment is -0.5395 and it involves medium dose iloprost. Interestingly, the synergy metric indicates that IP receptor activation by iloprost, a prostacyclin mimetic, was a more potent inhibitor of calcium mobilization compared to that observed with the activation of guanylate cyclase via GSNO release of NO (**Fig. 3F**). This was confirmed by the calculation of GSNO and iloprost percent inhibitions of various agonists used in this assay (S1 Table). It is also interesting to note that low and medium dose of GSNO slightly potentiated the medium dose thrombin-induced calcium release (15.73% and 13.32% increase respectively), consistent with previous findings that low levels of the NO donor sodium nitroprusside slightly potentiated thrombin-induced calcium release via store-operated calcium entry (SOCE), whereas higher levels inhibited thrombin-induced increases in calcium [17]. Similarly, in our assay, high dose GSNO inhibited the calcium release of medium dose thrombin (39.11% inhibition).

The maximum synergy score for the PAS experiment involved a high dose of convulxin and medium dose of thrombin (S_ij_ = 0.3461); the maximum of the trinary experiment involved medium dose convulxin, thrombin and GSNO (S_ijk_ = 1); the maximum of the higher order combination experiment involved high dose ADP, medium dose thrombin, high dose U46619 and low dose GSNO (S = 0.1085).

In fact, four of the five strongest positive pairwise synergies involved convulxin and thrombin (the fifth was medium dose thrombin and U46619). Of the five strongest positive synergies in the trinary experiments, medium dose convulxin and thrombin were involved in all of them, low and medium dose GSNO was involved in two of them. Of the five strongest positive synergies in the higher order experiments, thrombin and U46619 were present in four of them; low and medium dose GSNO is involved in four of them, convulxin and thrombin were present in two of them. It is apparent that convulxin and thrombin used together gave the strongest synergistic effects, thrombin and U46619 used together also accounted for some of the strongest synergistic effects. For the trinary and higher order experiments, the presence of low and medium dose GSNO was also implicated in the highest synergy scores, and only when thrombin was present, which supports the previous finding that low levels of NO potentiates thrombin-induced calcium release via SOCE [17].

The synergistic effects between convulxin and thrombin were similarly found by Keuren et al. [9] and it was thought that thrombin-mediated influx of platelet extracellular calcium (through PAR1 but not PAR4) enhances the collagen induced procoagulant response. This may explain why the synergistic effects between convulxin and the PAR1 agonist were not as prominent in previous PAS work that was done in the absence of extracellular calcium [1]. Another study that found similar synergistic effects between sub threshold concentrations of thrombin and GPVI showed that the synergism was independent of Src kinases and Syk [10]. As previously observed with EDTA-treated PRP activated by PAR1 agonist peptide and U46619 [2], thrombin activation of PAR1/PAR4 and the thromboxane mimetic U46619 were synergistic (S_ij_ = 0.1127 at medium dose of thrombin and low dose U46619), and especially at medium doses of thrombin and U46619 (S_ij_ = 0.1766).

### Inhibition of calcium release by iloprost

Iloprost and GSNO when used alone or together with each other had no effect on platelet calcium (top 9 binary conditions of **Fig. 3A-C**), as expected. However, these compounds elevate cAMP and cGMP to attenuate calcium mobilization [18–20] and this inhibition was clearly seen in the calcium traces (**Figs. 5, 7**).

Iloprost was a potent, rapid, and sustained inhibitor of convulxin activity (99.6–99.7% inhibition overall) (**S4B, S4H Fig.**), indicating that IP signaling was more rapid than GPVI signaling. Furthermore, iloprost was more potent than GSNO in inhibiting activity of all agonists this assay, for example, medium dose GSNO caused only 18.6% inhibition of convulxin activity (S1 Table). Convulxin caused slow platelet activation since it must multimerize GPVI to induce signaling [21]. During thrombin activation of PAR1/4, the inhibition by iloprost was also rapid (as seen by the offset in peak calcium levels), but was incomplete initially and became more pronounced after approximately 25 seconds post-stimulation (**S4D, S4J Fig.**). Low and medium levels of iloprost (0.1 and 1 x EC_50_) resulted in similar inhibition of thrombin calcium release (∼76.3 to 78.9% inhibition (**S4D, S4J Fig.**). In the experiments with thrombin, iloprost may have a diffusive and kinetic advantage over thrombin which must cleave PAR1/4 whereas iloprost simply must the bind IP receptor. A similar pattern was observed for ADP (**S4M, S4N Fig.**). However, there was no offset in peak calcium levels, potentially due to similar diffusive and binding kinetics of these two small molecules for their respective receptors. The inhibition by iloprost of ADP signaling only began after ADP-induced calcium level peaked around 20 seconds post-stimulation (41.5% to 71.7% inhibition at 0.1 and 1 x EC_50_, respectively). During U46619 stimulation of TP receptor, the inhibition by iloprost was apparent immediately after dispense and increased after calcium levels peaked (∼ 20 sec post-stimulation). Iloprost was slightly more potent against U46619 compared to ADP, causing 87.4% to 91.6% inhibition at 0.1 and 1 x EC_50_, respectively (**S4F, S4L Fig.**). Overall, iloprost was more fast-acting and potent against the slower signaling agonists such as convulxin (99.6–99.7% inhibition) or thrombin (∼76–79% inhibition) that required receptor multimerization or enzymatic cleavage, compared to small molecules that rapidly equilibrated with their receptors such as U46619 (87–92% inhibition) or ADP (41–72% inhibition). Iloprost may be less active against ADP compared to U46619 since ADP also binds the P_2_Y_12_ receptor which antagonizes cAMP pathways (**Fig. 1A**).

When ADP and convulxin were used simultaneously, ADP signaling dominated early calcium mobilization while convulxin signaling maintained sustained calcium levels. (**S4A, S4G Fig.**) With combined ADP/convulxin stimulation, medium dose iloprost resulted in only 71.1% inhibition (**S4G Fig.**) since it was not a complete blocker of the early signaling (at t < 30 sec) induced by ADP. A similar trend occurred with thrombin/convulxin co-stimulation (**S4C, S4I Fig.**), however, iloprost was more effective in this case (83.5% inhibition with medium dose iloprost, **S4I Fig.**) since thrombin/convulxin co-stimulation elevated calcium relatively slowly. When the weaker agonist U46619 (compared to ADP) was used with convulxin, iloprost remained a very potent inhibitor (98.9% inhibition with medium dose iloprost, **S4E, S4K Fig.**).

### Range of individual neural network prediction and donor responses

The range (i.e. intradonor variation) of individual NN predictions over 10 to 12 donors for single and pairwise agonist conditions (**S5 Fig.**) was comparable to the experimental observations, as expected for NNs trained exactly on those conditions (**S5A, S5B Fig.**). Similarly, the range of NN predictions for trinary agonist conditions matched the range for the experimental values as well (**S5C Fig.**). The range of the higher order NN predictions (≥ 4 agonists) was somewhat larger than the range observed in the corresponding individual experiments (**S5D–S5F Fig.**). Clearly for ≥ 4 agonists, the signaling pathways span a very complex platelet biology beyond the dimensionality of the pairwise training data. Nonetheless, the range of the NN predictions reflected to a large degree the range of the experiment itself. For example, the range of the NN-predictions in **S5E Fig.** was smaller than the range in **S5D Fig.**; the same trend was reflected in the range of the actual experiments. Furthermore, for ≥ 4 agonists, the experimental data comprises seven experiments spanning five donors, whereas the NN used in training spanned 12 donors, which in part explains why simulation ranges were larger than ranges of experimental observations. The NN range over all 120 NNs in **S5D Fig.** was larger than the experimental ranges, potentially reflecting donor variation but more importantly reflecting the difficulty of predicting higher dimensional responses. Despite the substantial range of individual NN predictions for the four-agonist condition depicted in **S5D Fig.**, the mean of the NN predictions predicted the mean response of the actual experiments, a benefit of the NN-ensemble approach for predicting a pooled population dynamic.

## Discussion

While the full complexity of receptor mediated signaling in platelets extends well beyond the known pathways indicated in **Fig. 1A**, a top-down approach using pairwise agonist scanning (PAS) provides an efficient data-driven method to predict platelet function. By using data obtained from multiple donors and training multiple NNs for each donor, a NN-ensemble (**Fig. 2**) allowed accurate prediction of 135 binary stimulations and 135 synergy parameters (**Fig. 3**). The synergy vector composed of the 135 synergy parameters is an experimentally measured human platelet phenotype (healthy adult male and female) that is fully predicted by the NN-ensemble. Furthermore, the NN-ensemble provided suitable prediction beyond the binary training set to predict trinary responses (**Figs. 4–5**), higher ordered responses (**Figs. 6–7**), and response to sequential stimuli (**Figs. 8–9**).

The major components of hemostasis and thrombosis that regulate platelet activation state are now quantitatively captured in the NN-ensemble. For large scale simulation of blood function, a user may specify or calculate any combination of the six agonists at different concentrations to produce a dynamic platelet calcium response representative of a healthy human donor. The NN is able to do this mainly because individual and pairwise interactions dominate platelet calcium signaling crosstalk in this assay, and because all single agonists are sampled across each of their full dose ranges [2]. An ordinary differential equation (ODE) model describing the calcium mobilization mediated by all six PAS agonists would likely require an estimated >500 kinetic parameters, many of which are unavailable [2].

From the perspective of a data-driven and top-down approach, NNs have proven quite robust and well matched to PAS data sets. Also NNs are ideal for multiscale simulations that involve crosstalk between receptors. However, NNs are not mechanistic models for identification or quantification of basic biochemical mechanisms. To facilitate future mechanistic model building, the full calcium data set is provided (S1 Dataset). For example, future mechanistic models of receptor signaling and crosstalk should be testable against the 135-parameter synergy map we measured. Such mechanistic models should account for RGS proteins, PKC, cAMP/PKA, cGMP/PKG, and phosphodiesterase pathways, as well as receptor desensitization pathways including ITIM/Shp2 phosphatase, receptor internalization, and receptor shedding pathways, along with regulation of store operated calcium entry. Improved predictive capability is not necessarily an outcome of constraining of NN nodes and linkages to a preconceived reaction topology (**Fig. 1**).

The calcium experiments that the NN was trained on included the contributions of SOCE because Apixaban was used in place of a calcium chelator as an anticoagulant. The effect of autocrinic effects by ADP and thromboxane secretion, however, were not significant in the PAS assay due to the dilute conditions of the assay (**S3 Fig.**). Such autocrinic effects however are naturally captured in multiscale simulations that include convection-diffusion of soluble agonists [6]. Other important inside-out signaling downstream of calcium mobilization, such as integrin engagement, granule release, shape change, and phosphatidylserine exposure can be simulated by incorporating the calcium model into a larger fine or coarse-grain model. For example, in Flamm et al. (2011), NN were trained via PAS using calcium-containing PRP and then used to predict platelet deposition rates on collagen in the absence of thrombin by accounting for platelet signaling in response to laboratory analogs of collagen, ADP, thromboxane, and prostacyclin [6]. Alternatively, the PAS methodology has also been adapted by Jaeger et al. [22] for flow cytometry instead of calcium measurements, so as to quantify inside-out signaling events such as: integrin α(IIb)β(3) activation, P-selectin exposure, and PS exposure using PAC-1, anti-P-selectin antibody, and annexin V, respectively.

The NN ensemble trained only on pairwise data was able to predict the calcium output of higher order agonist combination experiments with reasonably high accuracy. This was potentially due to a sparsity-of-effects principle: a system is largely dictated by main effects and lower order interaction [23]. Adding trinary conditions to the training data might theoretically improve prediction accuracy when it comes to higher order combinations. The improvement in accuracy should be a measure of the information content of trinary data. In separate studies, we tested the utility of adding trinary stimulation data to the PAS training set in order to enhance the predictive capability of the NN-ensemble. Trinary data were incorporated into the NN ensemble in 3 different ways, each time controlling for the number of NNs in the ensemble. The *first* set of trinary data collected comprised 160 trinary combinations of all six agonists at only low and medium concentrations, repeated on 10 donors. Adding this trinary data reduced the higher-order combination prediction accuracy from R = 0.824 to R = 0.784, and synergy score accuracy decreased from 0.66936 to 0.66145. The *second* set of trinary data collected comprised 27 combinations of only three agonists (ADP, convulxin, U46619) in the full dose range, repeated on eight donors. Adding this trinary data did not substantially affect the prediction accuracy (calcium trace accuracy from R = 0.810 to R = 0.806, and synergy correlation from R = 0.66367 to R = 0.68387). The *third* set of trinary data collected was an unbiased, random sampling (n = 54) of the complete trinary space done on a single donor, who was not part of the original PAS dataset. Adding this trinary data increased prediction accuracy of the time courses (from R = 0.871 to R = 0.906), but reduced the accuracy of synergy scores prediction from R = 0.6953 to R = 0.53925. We conclude that incorporating a randomly sampled trinary dataset can moderately increase prediction accuracy, whereas adding a biased sample of the trinary space does not increase accuracy. In fact, adding a sample of the trinary space that does not span the full dose range may reduce accuracy. However, even in the best of the three scenarios tested, adding trinary data to the PAS training dataset did not substantially improve accuracy.

The 120-NN ensemble (R = 0.87134, mean-squared error MSE = 0.0129) was more accurate than the average individual NN within the ensemble (R average = 0.6566, MSE average = 0.0501) in predicting the calcium traces of higher order combination experiments. Even though the most accurate individual NN in the 120-NN ensemble (R = 0.91391, MSE = 0.0092) was more accurate than the ensemble itself, its prediction output was significantly noisier, and its synergy score prediction accuracy was much lower (R = 0.6953 to R = 0.4609). The ensemble approach reduced variances in prediction output, increased accuracy above the average NN in the 120-NN ensemble, consistent with previous findings [24], and is generally thought to be more robust [25–28]. Ensemble pruning is often used to eliminate individual models from the ensemble based on certain criteria so as to improve the new ensembles predictive ability [28]. Using the interquartile range (IQR) outlier detection method, eight high outliers spanning seven different donors were identified in the mean-squared error (MSE) measurements of each of the 120 individual NNs. Removing the eight most inaccurate individual NNs from the ensemble did not improve the ensemble accuracy (R-value improved slightly from 0.87134 to 0.87768, but MSE increased slightly from 0.0129 to 0.0148, synergy score R decreased slightly from 0.6953 to 0.68632, and noise in ensemble predictions increased). Diversity of the models (in this case originating from the different random initial weights generated at the beginning of each NN training) comprising an ensemble is important for accuracy and robustness [29–31]. To increase diversity, heterogeneous ensembles may be used instead; for example, changing the training parameters of select individual NNs or incorporating into the NN-ensemble regression models other than NNs [28].

We found that the NN-ensemble was able to account for the dynamics and magnitude of one pathway relative to that of another. Iloprost was a very potent antagonist in this assay. In vivo, prostacyclin activates IP to increase cAMP and cGMP-dependent protein kinases pKA and pKG. Both pKA and pKG phosphorylate RGS18 (a G-protein regulator), which eventually turns off G_q_-signaling, the main activation pathway for U46619 and thrombin (via PAR-1) [32]. ADP signaling through P_2_Y_1_ also goes through G_q_-signaling. However, ADP also signals through the P_2_Y_12_ receptor, which involves the G_i_ protein (**Fig. 1A**).Thrombin signaling through PAR4 can go through either G_q_ or G_i_. The existence of alternate signaling routes (through G_i_) explain why iloprost inhibition is markedly less potent for ADP, and slightly less effective for thrombin as well. Furthermore, signaling through the G_i_ protein inhibits the rise in adenylate cyclase (precursor of cAMP which decreases intracellular calcium levels). As expected from studies of “coated” platelets [33] and studies with similar findings [9,10], convulxin and thrombin were quite synergistic in the PAS assay. Additionally, thrombin and TP receptor signaling were somewhat synergistic, consistent with previous findings [2,6]. In future work, the NN-ensemble can be incorporated in multi-scale and hierarchical simulations of bleeding or clotting with linkages to vascular pathophysiology. While exogenously added thrombin was used in PAS, not all of this thrombin may reach the platelet due to antithrombin. This may right shift the thrombin potency [34]. Iloprost and GSNO were also included in this assay to recapitulate endothelial-derived prostacyclin and nitric oxide effects on platelet function. The development of a healthy human platelet calcium calculator can enable various applications such as predicting thrombosis or hemostasis under flow condition or extracting information from in vitro diagnostics, potentially using platelets from patients with cardiovascular disease risks.

## Supporting Information

S1 Fig Pairwise Agonist Scanning experimental method. Platelet-rich-plasma was obtained from a healthy donor and incubated with Fluo 4-NW calcium dye (Invitrogen) for 30 minutes, then dispensed into a 384-well plate called the “read plate”. At the same time, a high-throughput liquid handler (PerkinElmer Janus) assembled another 384-well plate containing all combinations of six agonists at three doses called the “agonist plate”. Molecular Devices FlexStation 3 auto-dispenser and microplate reader measures calcium fluorescence in the read plate for 260 seconds. The FlexStation 3 dispensed contents from the agonist plate into the read plate at the 20 second mark. This experiment was done in duplicates with ten donors (five male and five female).
(EPS)

S2 Fig Dose response curves of each agonist. This set of dose response curves were obtained for a single donor done in quadruplicate, and is representative of the dose response curves for the total of six donors used to calculate the average EC_50_ values shown in the table inset. EC_50_ values were calculated by fitting a four-parameter hill function curve (dashed lines) to the area under curve of the baseline-normalized fluorescence intensity (F/F_0_) calcium time course. For the dose response of platelet antagonist iloprost, platelets were simultaneously stimulated with PAR1 agonist SFLLRN (40μM). For the dose response of platelet antagonist GSNO, platelets were simultaneously stimulated with 1μM ADP.
(EPS)

S3 Fig Investigation of autocrinic signaling effects. To determine whether or not significant secondary autocrinic amplification effects by ADP and thromboxane secretion were present in the PAS assays, apyrase (ADP hydrolyzing enzyme, 2 Units/ml) and indomethacin (COX-inhibitor, 15μM) were used. GSNO, Iloprost, U46619, thrombin and convulxin at 0.1, 1, or 10 X EC_50_ were added to platelets in similar conditions as in the PAS experiments (12% PRP, 250nM Apixaban). In the case of the inhibitors GSNO and Iloprost, platelets were co-stimulated with 60mM SFLLRN, a PAR1 activator. Only one of the 60 conditions tested with added inhibitors produced a detectable reduction in calcium signal (one-tailed T-test P < 0.05).
(EPS)

S4 Fig Analysis of iloprost inhibition effects. Data from PAS and trinary combination experiments also provided insight into the inhibitory effects of iloprost on other agonists. **(B, H)** Iloprost was a potent and sustained inhibitor of GPVI-induced calcium release (99.6% and 99.7% inhibition by low and medium dose iloprost respectively). Iloprost was a moderately potent inhibitor of **(D, J)** thrombin activity (76–79% inhibition) and **(F, L)** U46619 activity (87–92% inhibition). **(M, N)** Iloprost was least effective on ADP (41–72% inhibition). **(A, G)** With combined ADP/convulxin stimulation, low and medium dose iloprost resulted in only 61% and 71% inhibition respectively. **(C, I)** With thrombin/convulxin co-stimulation, however, iloprost was more effective (75%–84% inhibition). **(E, K)** When the weaker agonist U46619 (compared to ADP) was used with convulxin, iloprost remained a very potent inhibitor (95%–99% inhibition).
(PDF)

S5 Fig Range of individual neural network responses and donor responses. The range of the NN predictions reflected to a large degree the range of the experiment itself. **(A-C)** The range of single **(A)**, binary **(B)** and trinary **(C)** predictions matched almost exactly the range of its corresponding experiments. **(D-F)** Although the range of the higher order NN predictions (≥ 4 agonists) was larger than the range observed in the corresponding individual experiments, the mean of the NN predictions was a good fit of the mean response of the actual experiments, a benefit of the NN-ensemble approach for predicting a pooled population dynamic.
(EPS)

S6 Fig Neural network prediction of the trinary combination experiment. Experimental and NN-predicted calcium traces are plotted for all 160 trinary conditions (all single and trinary combinations of agonists at two concentrations: 0.1x EC_50_ and 1x EC_50_). Rescaled to 0.5 for easy visualization.
(PDF)

S1 Table Percent inhibition of medium dose iloprost and GSNO on medium doses of various agonists. Iloprost was a more potent inhibitor than GSNO on all the agonists in the PAS assays. Interestingly, medium dose GSNO slightly potentiates thrombin-mediated calcium mobilization.
(DOCX)

S1 Dataset Calcium data used in all experiments. To facilitate future building of a mechanistic platelet calcium model, the full calcium data set is provided. This dataset comprises MATLAB structures that contain dynamic calcium data in response to various combinations and permutations of up to six agonists used in PAS, trinary, higher order and sequential experiments. There are 24 PAS experiments spanning 12 donors, 10 PAS experiments spanning 10 donors, 7 higher order experiments spanning 5 donors, and 1 sequential addition experiment done for a single donor.
(ZIP)

S2 Dataset Trained neural networks (NNs). This dataset comprises all 120 NNs trained on 12 donor-specific PAS experiments (10 NNs trained per donor, PAS experiments averaged over 2 repetitions) Trained NNs are in the form of MATLAB networks that contain the final trained weights for all 12 nodes in the 2-layer NN configuration. The trained NNs may be used to make dynamic calcium predictions in response to all combinations and permutations of up to six agonists (ADP, CVX, thrombin, U46619, iloprost, GSNO).
(ZIP)

S1 Code Human Platelet Calcium Calculator. A MATLAB M-File that allows users to specify a combination or permutation of up to six agonists (ADP, CVX, thrombin, U46619, iloprost, GSNO) in the full dose range, and obtain the corresponding predicted dynamic calcium time trace based on the 120 NNs that were trained on PAS experiments. This code requires the trained NNs in **S2 Dataset** to run.
(ZIP)
