# Supplementary figures and images for: A Human Platelet Calcium Calculator Trained by Pairwise Agonist Scanning
Source: PLoS Comput Biol. 2015 Feb 27;11(2):e1004118. doi: 10.1371/journal.pcbi.1004118 (PMC4344206; doi:10.1371/journal.pcbi.1004118)

# S4

## Iloprost 0.1 x EC<sub>50</sub>

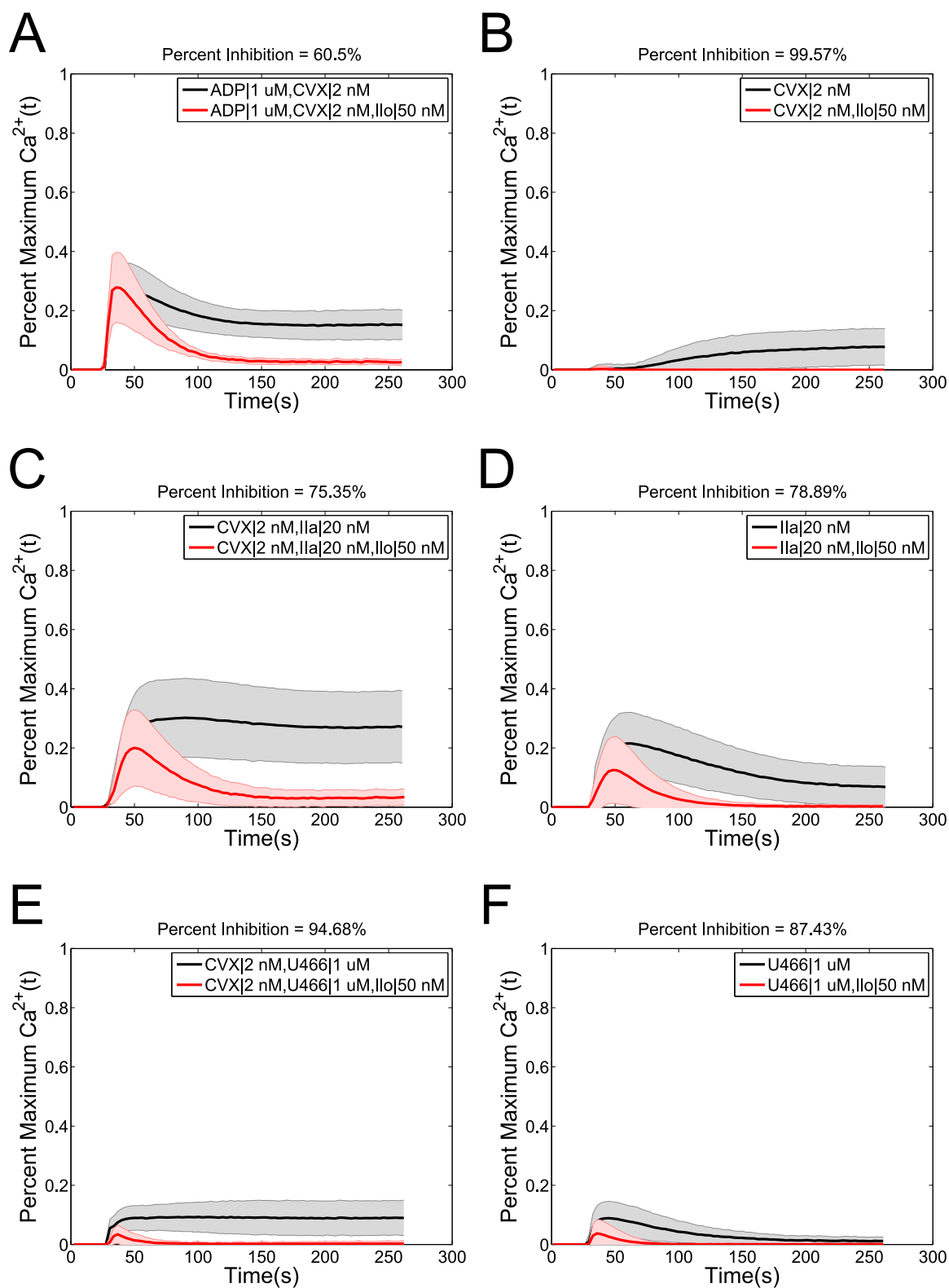

S4

Iloprost 1 x EC<sub>50</sub>

G

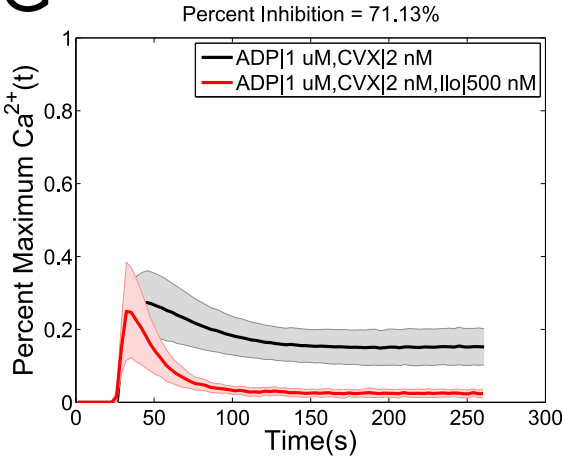

H

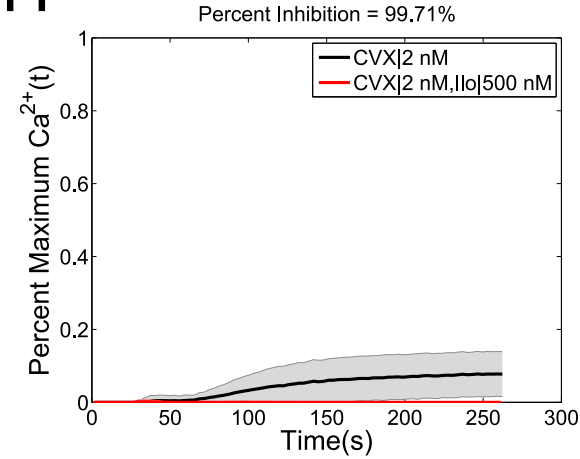

I

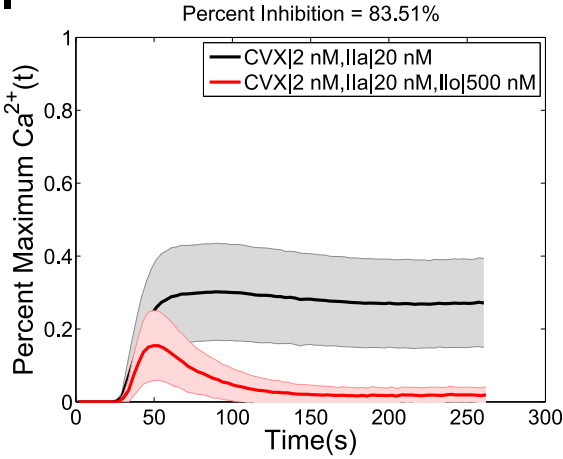

J

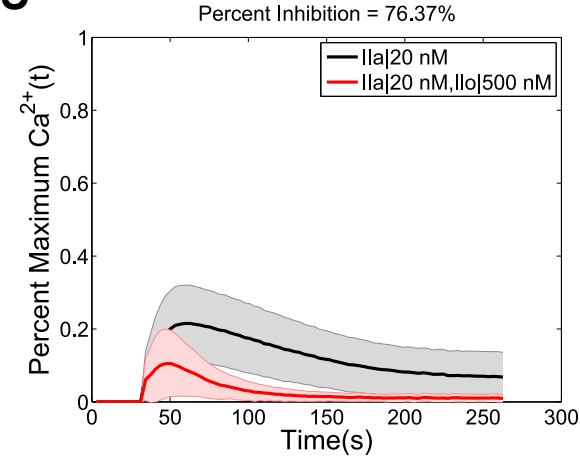

K

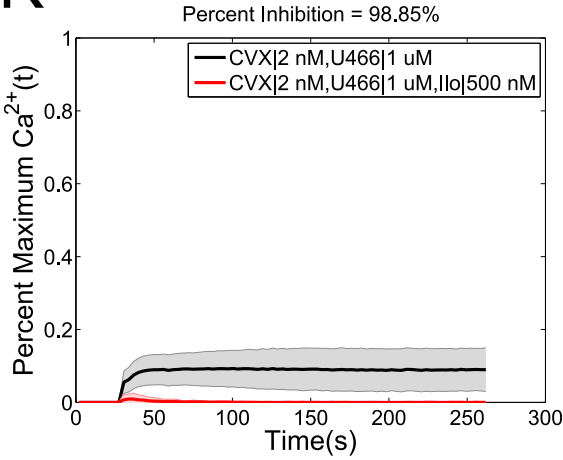

L

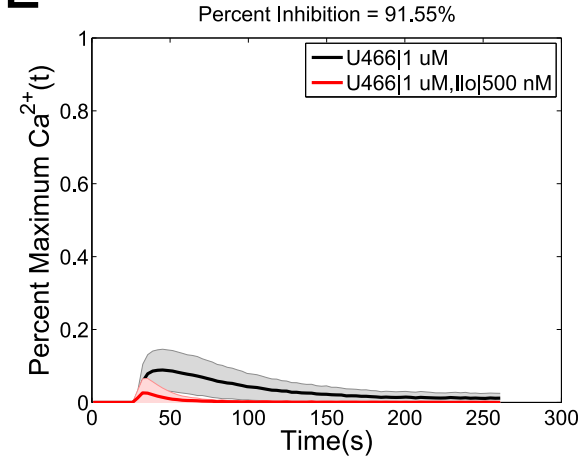

S4

ADP

M

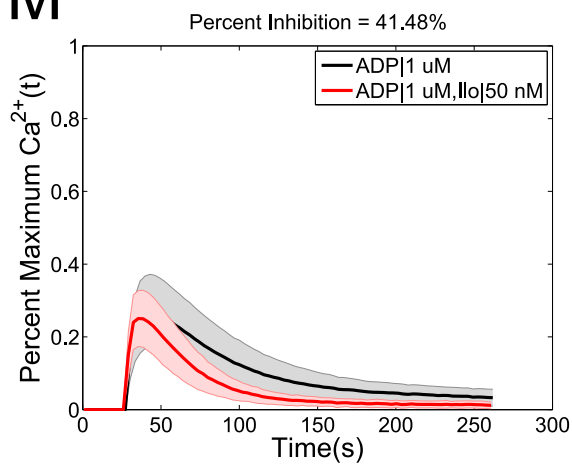

N

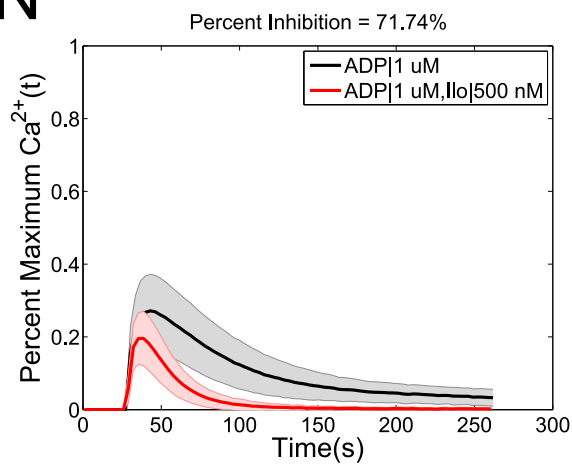

Supplement: S4 Fig — Data from PAS and trinary combination experiments also provided insight into the inhibitory effects of iloprost on other agonists. (B, H) Iloprost was a potent and sustained inhibitor of GPVI-induced calcium release (99.6% and 99.7% inhibition by low and medium dose iloprost respectively). Iloprost was a moderately potent inhibitor of (D, J) thrombin activity (76–79% inhibition) and (F, L) U46619 activity (87–92% inhibition). (M, N) Iloprost was least effective on ADP (41–72% inhibition). (A, G) With combined ADP/convulxin stimulation, low and medium dose iloprost resulted in only 61% and 71% inhibition respectively. (C, I) With thrombin/convulxin co-stimulation, however, iloprost was more effective (75%–84% inhibition). (E, K) When the weaker agonist U46619 (compared to ADP) was used with convulxin, iloprost remained a very potent inhibitor (95%–99% inhibition). (PDF) [file pcbi.1004118.s004.pdf]

S6

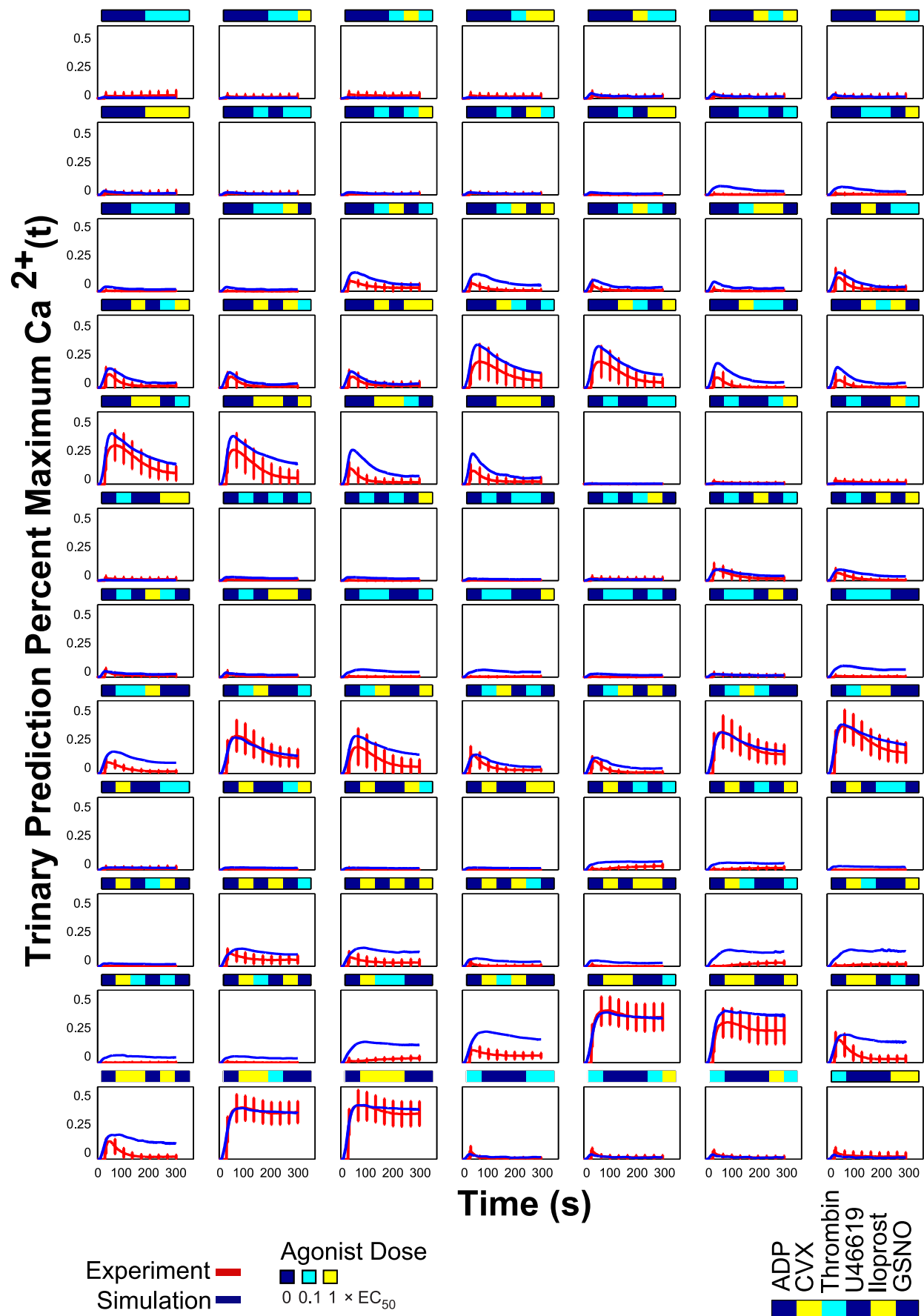

S6

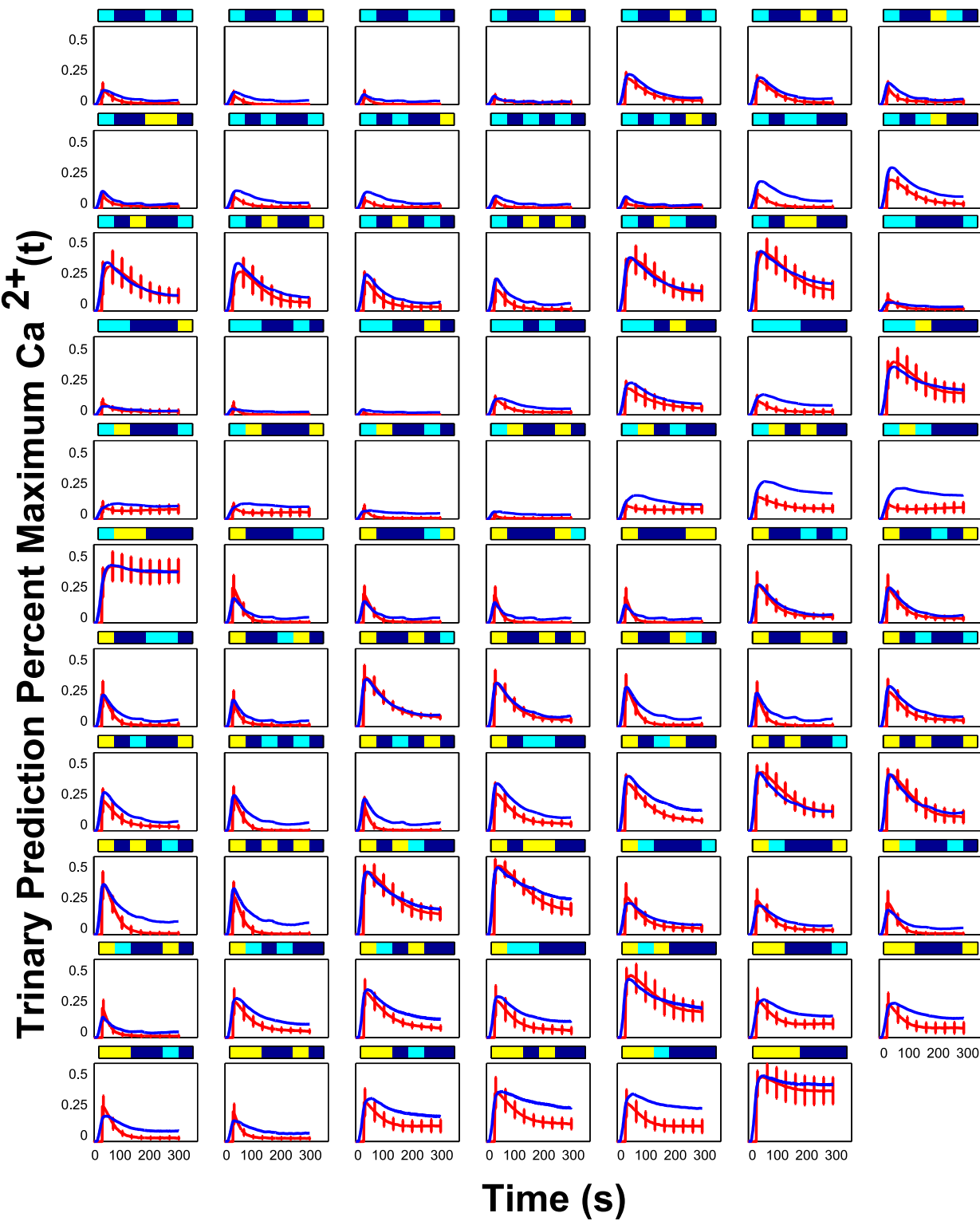

Experiment — Simulation —

Agonist Dose  
0 0.1 1  $\times \text{EC}_{50}$

ADP  
CVX  
Thrombin  
U46619  
Iloprost  
GSNO

Supplement: S6 Fig — Experimental and NN-predicted calcium traces are plotted for all 160 trinary conditions (all single and trinary combinations of agonists at two concentrations: 0.1x EC50 and 1x EC50). Rescaled to 0.5 for easy visualization. (PDF) [file pcbi.1004118.s006.pdf]
