# Supplementary material for: A Human Platelet Calcium Calculator Trained by Pairwise Agonist Scanning
Source: PLoS Comput Biol. 2015 Feb 27;11(2):e1004118. doi: 10.1371/journal.pcbi.1004118 (PMC4344206; doi:10.1371/journal.pcbi.1004118)
Supplement: S1 Table — Iloprost was a more potent inhibitor than GSNO on all the agonists in the PAS assays. Interestingly, medium dose GSNO slightly potentiates thrombin-mediated calcium mobilization. (DOCX) [file pcbi.1004118.s007.docx]

**Supporting Information Tables**

**Table S1. Percent inhibition of medium dose iloprost and GSNO on medium doses of various agonists**

|  | **% inhibition by Iloprost** | **% inhibition by GSNO** |
| --- | --- | --- |
| **ADP** | 71.74% | 34.08% |
| **CVX** | 99.71% | 18.58% |
| **Thrombin** | 76.37% | -13.32% |
| **U46619** | 91.55% | 67.09% |

Iloprost was a more potent inhibitor than GSNO on all the agonists in the PAS assays. Interestingly, medium dose GSNO slightly potentiates thrombin-mediated calcium mobilization.
